# Psychometric properties and population norms of the positive mental health instrument in a representative multi-ethnic Asian population

**DOI:** 10.1186/s12874-018-0487-9

**Published:** 2018-03-15

**Authors:** Janhavi Ajit Vaingankar, Mythily Subramaniam, Linda Wei Lin Tan, Edimansyah Abdin, Wei Yen Lim, Hwee Lin Wee, Siow Ann Chong, Rob Martinus van Dam

**Affiliations:** 1 Institute of Mental Health, 10, Buangkok View, Singapore, 539747 Singapore; 2 Saw Swee Hock School of Public Health and Department of Medicine, Yong Loo Lin School of Medicine, National University of Singapore and National University Health System, Singapore, Singapore; 3 Lee Kong Chian School of Medicine, Nanyang Technological University, Singapore, Singapore; 4 Department of Clinical Epidemiology, Tan Tock Seng Hospital, Singapore, Singapore

**Keywords:** Confirmatory factor analysis, General population, Normative data, Reliability, Validity

## Abstract

**Background:**

Measures of mental well-being and positive mental health (PMH) have been largely developed and used in Western populations, however, data on representative Asian communities are lacking. Using data from a population sample, this study sought to establish psychometric properties and norms of the PMH Instrument (PMH-I), a measure of positive mental health developed in Singapore.

**Methods:**

We conducted a nationally representative survey among 1925 adults aged 18–79 years of Chinese, Malay, Indian or other ethnicity. Participants reported socio-demographic characteristics and completed the PMH-I along with measures of health-related quality of life (HRQoL) and psychological distress. Construct validity of the PMH-I was assessed using confirmatory factor analysis and concurrent validity was tested through correlation with other psychological measures. Normative PMH values and differences in population subgroups were estimated.

**Results:**

The six-factor-higher-order structure of the PMH-I comprising six subscales of general coping, emotional support, spirituality, interpersonal skills, personal growth and autonomy and global affect was confirmed. Concurrent validity was shown through significant positive correlation of the total PMH score and its subscales with HRQoL and an inverse correlation with psychological distress. Weighted age, gender and ethnicity-specific norms were derived for the Singapore population. Total PMH was significantly higher in participants aged over 40 years as compared with 18–29 year olds and in non-Chinese ethnic groups as compared with Chinese. These differences were observed for all PMH-I subscales, with the exception of emotional support and interpersonal skills score differences by age. In contrast, gender, marital status, and education level were significantly associated with some of the subscales, but not with total PMH.

**Conclusions:**

These results support the psychometric properties of the PMH-I in a multi-ethnic Asian population sample. The generalizable population-based norms support the application of the PMH-I for measuring mental health and assessing its determinants within the Singapore general population.

**Electronic supplementary material:**

The online version of this article (10.1186/s12874-018-0487-9) contains supplementary material, which is available to authorized users.

## Background

Mental health is inherent to all individuals and is globally accepted as a determinant of overall health, well-being, and resilience [1]. There is also a broad consensus on mental health being ‘more than just absence of mental illness’ [1]. Several conceptual theories exist that propose a framework for multiple distinct but related aspects of mental health [2, 3]. Two prominent approaches that have shaped the expansion of this field are a focus on hedonic or subjective well-being and the other on eudemonic or psychological well-being [2, 4]. While the former relates to striving for positive experiences, the second emphasizes the role of psychological needs and self-actualization in well-being. The World Health Organization (WHO) operationalized a working definition for mental health as a ‘state of well-being’ in which individuals realise their own potential, are able to cope with stress, and contribute to the community [1]. This definition provided a composite of the two well-being approaches and has served as a broad theoretical opportunity to investigate mental health status of communities across the world.

Consequently, understanding how mental health influences long-term well-being and using these insights to develop interventions to prevent a decline in overall health has gained momentum as a focus area in public health research [1, 5–8]. Longitudinal studies have shown that high levels of psychological well-being serve as a protective factor against mental illnesses and psychopathology [9] and account for reduction in the risk of mood and anxiety disorders [10]. In addition, positive affect and emotional well-being may protect against disability in old age [11]. Mental well-being has also been associated with biological markers of physical health, better hormonal regulation, and a lower risk for chronic diseases and mortality [12, 13]. This growing evidence on mental well-being as a determinant of better psychological and physical health has further increased interest in interventions to improve mental well-being of individuals and populations as a whole [14]. Such initiatives require valid, reliable and generalizable estimates of mental health that go beyond the mere absence or presence of psychological distress and help in tracking population mental health status [15, 16].

Measuring mental health and well-being in individuals provides unique information on emotional and social well-being of a person, which is not provided by using psychiatric assessments alone. However, with the lack of a single universally accepted definition of mental well-being [3], a number of scales, including the Satisfaction With Life Scale [17], Psychological Well-being Scale [18], Affectometer [19], Short Form (SF)-36 [15], WHO-Five Well-being Scale [15], and the Warwick Edinburgh Mental Well-being Scale (WEMWBS) [20] have been used to assess different components of mental well-being.

More recently, the Positive Mental Health Instrument (PMH-I) and the Positive Mental Health Questionnaire (PMHQ) were developed in multi-ethnic Asian and European populations, respectively [21, 22]. There are several similarities between these two measures. Both measures were developed using mixed methods [21, 23] and present a multi-dimensional structure of PMH comprising six factors relating to hedonic and eudemonic constructs of mental well-being [3]. While the PMH-I has 47 items belonging to the domains of general coping, emotional support, spirituality, interpersonal skills, personal growth and autonomy, and global affect, the PMHQ has 39 items representing personal satisfaction, prosocial attitude, self-control, autonomy, problem-solving and self-actualization, and interpersonal relationship skills. The differences lie in their conceptual and psychometric approach and the characteristics of the populations used for the development and validation of these measures. The PMHQ was based on the Multifactor Model of positive mental health [22] that proposes ‘close inter-relationship between physical and mental health’ and the initial development of the tool was conducted in people with chronic physical health problems. Although it has since been validated in university professors and students in Europe [23, 24], to the best of our knowledge, it has not been validated among the general population or different ethnic groups.

The PMH-I was developed to assess the level of positive mental health in the multi-ethnic adult Asian population in Singapore using mixed methods guided by the theory of mental health being more than the absence of mental illness [1] and with an emphasis on only the positive aspects of mental well-being [21, 25]. Its structure and psychometric properties have been established in a general population sample and confirmed among service users with mental disorders [21, 26]. However, the previous studies were conducted in smaller samples, excluded participants who were not of Chinese, Malay and Indian ethnicity, and adopted convenience sampling to recruit participants, thereby limiting the representativeness of the findings. Because of the selective nature and size of the investigated samples, evidence supporting the structure and validity of the scale could not be generalized to the general population.

The present study therefore aimed to validate the PMH-I in a representative urban Asian population sample to establish its psychometric properties by analyzing dimensionality, reliability and concurrent validity of the PMH-I. We hypothesized that the PMH-I would fulfil the factor loading and fit requirements of a multi-dimensional six-factor-higher-order measure [21]. In addition, we expected that the PMH-I score would be positively associated with health related-quality of life (HRQoL) and inversely associated with psychological distress. The study also examined socio-demographic factors including age, gender, ethnicity, marital status and education level as determinants of PMH and its domains. In addition, the study aimed to generate normative values of the PMH-I to allow comparisons between different age, gender and ethnic groups in the population.

## Methods

### Ethics

Ethical approval was obtained from the National University of Singapore Institutional Review Board (NUS-IRB) prior to the start of the study (NUS IRB, reference 13–512). Written informed consent was obtained from all adult participants. For participants aged 18 to 20 years, written consent was also obtained from their parent or legal guardian. Strict confidentiality was maintained for participant’s identifiers and test results by applying de-identified collection of data, password protected data recording, and aggregate data analysis.

### Study design

This study on the PMH-I used data collected in the Singapore Health (SH) - 2 study, which was a cross-sectional study conducted between April 2014 and March 2015 to assess mental and physical health and lifestyle behaviours in a nationally representative general population sample of Singapore residents aged between 18 and 79 years residing in the west, north, north-east and south-eastern central zones in Singapore. The SH-2 study collected comprehensive data from participants using self-report measures and a health screening.

### Setting, sample selection and field work

The National Database on Dwellings sampling frame maintained by the Department of Statistics, Singapore had addresses of 32,100 households in 16 geographically clustered areas having at least one resident aged 15 to 79 years. The areas were clustered in the vicinity of four clinics in the survey zones that served as health screening sites for conducting laboratory investigations for the SH-2 study. A total of 15,000 household addresses were randomly selected from the sampling frame. A notification letter was sent to these addresses informing residents of the survey and the enumeration exercise that included a telephone number to contact the team for any queries. Trained enumerators then conducted the enumeration visit two weeks from the notification letter being sent out to identify all household members who met the inclusion criteria – being Singaporean or Permanent Resident, aged 18 to 79 years at the time of enumeration, residing at the address for at least four days each week and staying in the household for the next 3 months or longer. Household members who were bedridden or wheelchair bound, had severe mental retardation or mental illness, permanent disability, stroke or injury resulting in loss of speech, and pregnant women were excluded from the survey. For each household, one eligible resident was selected using Kish selection grid [27] and invited to participate in the study. A trained interviewer then contacted the selected household member to invite him/her to participate in the study. After obtaining written consent from the participant, interviewers completed the SH-2 survey questionnaire via face-to-face computer-assisted personal interviewing (CAPI).

### Data quality control

Survey responses were checked for missing values, data type errors and range sensibility using CAPI embedded logic checks and prompts with built-in algorithms whereby participants had to answer each question before moving on to the next one. All interviews were audio-recorded for quality control purposes. About 20% of the surveys and interviewer-specific records were randomly selected for verification of the responses against the audio-recording of the interviews. Data errors, inconsistencies and outliers were clarified through direct verification with the participants whenever necessary. The PMH-I was self-administered by the participants using CAPI in the presence of an interviewer who assisted them if they encountered any difficulties in completing the instrument.

### Survey questionnaire

A structured questionnaire was used in the survey to obtain information on the socio-demographic background, major non-communicable diseases and related risk factors, and general well-being of the participants. Survey questionnaires were pre-tested among local participants before initiating the survey. For the purpose of the current analysis, the following data and measures were used.

PMH-I [21]: The 47-item PMH-I includes six subscales: general coping (9 items), emotional support (7 items), spirituality (7 items), interpersonal skills (9 items), personal growth and autonomy (10 items), and global affect (5 items) (Additional file 1: Table S1). For the first five subscales, participants were asked to select a number showing how much the item describes them on a scale from 1 to 6, where ‘1’ represents ‘not at all like me’ and ‘6’ corresponds to ‘exactly like me’. The ‘Global affect’ subscale includes a list of five affect indicators and requires participants to indicate ‘how often over the past four weeks they felt – calm, peaceful, etc. using a 5-point response scale. The PMH-I comprises positively worded items, for example, ‘I try not to let it bother me’, ‘I try to get emotional support from family and friends’ and ‘I have confidence in the decisions I make’. Subscale and total PMH scores were obtained by adding scores of the respective items and dividing the scores by the number of items in each subscale, where higher scores indicate higher PMH. At the time of the survey, the PMH-I was available solely in English and was administered to only those participants who were literate in English.

European Quality of Life-5 Dimensions 5 levels (EQ-5D 5 L) [28]: The EQ-5D 5 L is a measure of HRQoL, developed by the EuroQol Group that measures five dimensions of health (mobility, self-care, usual activities, pain/discomfort and anxiety/depression), within five levels — corresponding to ‘no problems”, ‘light problems,’ ‘moderate problems,’ ‘severe problems’ and ‘unable/extreme problems’ — giving a total of 3125 unique composite health states. Responses to these five dimensions were converted into one of 243 unique EQ-5D health state descriptions, which range between no problems on all five dimensions (11111) and disability/extreme problems on all five dimensions (55555). EQ-5D Index scores were derived using the time trade-off values for the UK general population. Index scores range from − 0.594 to 1.00, with negative values representing health states worse than being dead, 0 representing being dead and 1.00 representing the state of full health. The EQ-5D also has a visual analogue scale (VAS) which indicates self-rated health using a 20 cm vertical VAS with endpoints labelled ‘the best health you can imagine’ denoted by ‘100′ and ‘the worst health you can imagine’ (‘0′). Participants were instructed to write the number marked in the scale on a box to indicate their health on the day of the interview.

Kessler 6 Psychological Distress scale (K6) [29]: The K6 is a widely used measure of psychological distress that consists of six self-report questions about frequency of depressive and anxiety symptoms in the past 4 week period on a five-point rating scale from ‘none of the time’ to ‘all of the time’. A total score was obtained that ranged from 6 (indicating no distress) to 30 (indicating severe distress).

Socio-demographic information on age, gender, ethnicity, marital status and education level were also collected during the survey.

### Response rate

A total of 2690 Singapore residents aged 18 to 79 years participated in SH-2, out of the 7743 eligible household members identified through the enumeration exercise, yielding a response rate of 35% for the SH-2, which is typical of door-to-door surveys in Singapore. Of the 2690 participants, 1925 participants who were literate in English and had completed the PMH-I, served as the study sample for this analysis.

### Sample weights

Sample weights were calculated for the household enumeration exercise and survey. For the household enumeration exercise, sample weights (WEE) comprised weights for unequal probability of selection and non-response computed based on dwelling type (public or private housings) and region of dwelling (16 postal districts). For the study fieldwork, sample weights (WSF) comprised weights for unequal probability of selection and non-response computed based on age, gender and ethnicity (Chinese, Malay, Indian and other ethnicities) of the residents. Post-stratification weights (WPS) were computed based on the age, gender and ethnicity with reference to the Singapore resident population at June 2014. The overall sample weights were the product of WEE, WSF and WPS.

### Statistical analysis

Statistical analyses were carried out using the SPSS and MPLUS software programs. The factor structure of the PMH-I was confirmed using confirmatory factor analysis (CFA). All items were treated as categorical variables. The CFA was conducted with MPLUS software using polychoric item correlations matrix with weighted least squares with mean-adjusted chi-square statistic (WLSM) estimator that provides estimates of item loadings and thresholds. Overall model fit was measured using a number of goodness-of-fit (GOF) statistics based on the following criteria: the comparative fit index (CFI), the Tucker-Lewis index (TLI), the root mean square error of approximation (RMSEA) and standardized root mean square residual (SRMR). Cut-off values suggested by Hu and Bentler were used: above 0.95 for TLI and CFI, below 0.05 for RMSEA and below 0.08 for SRMR [30]. Internal consistency reliability was estimated for each subscale with Cronbach’s alpha coefficient, with an acceptable level set at 0.7. Pearson correlation tests were used to establish the concurrent validity of the PMH-I and its subscales with other measures using known-group validity criteria based on previous studies [21, 27]– (i) Total PMH score will show significant low to moderate positive correlation with HRQoL (EQ-5D Index) and self-rated health (EQ-5D VAS), (ii) Total PMH score will show significant low to moderate negative correlation with psychological distress (K6 total score) and (iii) psychological distress will show strongest negative correlation with ‘Global affect’ domain. Floor and ceiling effects were calculated by identifying the proportion of participants that either had the lowest (1) or the highest (6) possible scores for the PMH total and subscale scores. Age, gender and ethnicity-specific norms were derived for the Singapore population and difference in their mean PMH total and subscale scores was identified through SPSS Complex Samples general linear regression models. Weighted main effects multivariable linear regression analysis was conducted after including age, gender, ethnicity, marital status and education level as covariates and total PMH and subscale scores as dependent variables to investigate the relation of the important population groups of interest with PMH. Two-sided statistical significance was set at *p* value of less than 0.05.

## Results

The socio-demographic characteristics of the participants are presented in Table 1. The mean age of the participants was 40.1 years. The sample comprised 71.1% Chinese, 14.1% Malays, 11% Indians, and 3.9% other ethnicities. This is similar to the ethnic distribution in the Singapore population of 74.2% Chinese, 13.3% Malay, 9.2% Indian and 3.3% other ethnicities. The majority of the sample were married (59.1%) and employed (71.9%).

**Table 1:** Socio-demographic characteristics of the sample (*N* = 1925)

		*n*	Weighted % (SE)	General population^a^ %
Age, years (Mean, SE)	40.1, 0.4			40.5
Gender	Men	921	52.1 (1.4)	49.3
	Women	1004	47.9 (1.4)	50.7
Ethnicity	Chinese	1149	71.1 (1.1)	74.2
	Malay	320	14.1 (0.9)	13.3
	Indian	366	11.0 (0.7)	9.2
	Other	90	3.9 (0.5)	3.3
Education level	Primary and below	70	3.0 (0.4)	
	Secondary to JC	810	40.0 (1.4)	
	Vocational or Polytechnic diploma	424	24.0 (1.2)	
	University and above	621	33.0 (1.3)	
Marital status	Never married	583	34.9 (1.4)	
	Married	1168	59.1 (1.4)	
	Separated /divorced /widowed	170	6.1 (0.6)	

^a^Singapore Census 2010

**Fig. 1:**
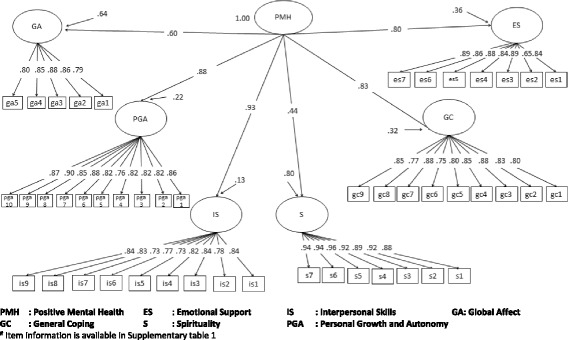
Factor structure of PMH-I^#^ in the Singapore general population

The CFA confirmed the six-factor-higher-order model of the PMH-I with fit indices fitting the thresholds (RMSEA = 0.047, CFI = 0.958, TLI = 0.95, SRMR = 0.043). The standardized loadings of the six-factors to the higher-order factor were high and ranged from 0.437 to 0.930. The factor loadings of the scale are illustrated in Fig. 1. The Cronbach’s alpha coefficient for the total PMH scale was 0.961. The alpha coefficients for general coping, emotional support, spirituality, interpersonal skills, personal growth and autonomy, and global affect subscales were 0.923, 0.898, 0.912, 0.958, 0.938, and 0.886, respectively. We also conducted a sensitivity analysis of the psychometric properties separately for the three major ethnic groups. Factor solutions fulfilled the set fit indices criteria [30] for these ethnicities (RMSEA, CFI, TLI: Chinese: 0.050, 0.955, 0.953; Malay: 0.045, 0.963, 0.961; Indian: 0.054, 0.941, 0.938, respectively; Additional file 1: Figure S1a-c).

Table 2 shows the Pearson’s correlation coefficient between the PMH-I, its subscales and concurrent validity measures. PMH subscales showed significant moderate to high correlations with the total PMH score, with values ranging from 0.573 to 0.849 (*p* < 0.01). The total PMH and subscales (except for the Spirituality subscale) showed significant positive correlations with the EQ-5D Index for HRQoL (r ranged from 0.100 to 0.254) and self-rated health on the EQ-5D visual analogue scale (VAS; r ranged from 0.139 to 0.299) and negative correlations with the K6 scale of psychological distress (r ranged from − 0.180 to − 0.416). The global affect subscale showed highest correlations with EQ-5D Index, EQ-5D VAS and K6 scores.

**Table 2:** Pearson’s correlation coefficients of the PMH subscales and concurrent measures

	Total Positive Mental Health	General Coping	Emotional Support	Spirituality	Interpersonal Skills	Personal Growth and Autonomy	Global Affect
Positive Mental Health	1						
General Coping	0.80^a^	1					
Emotional Support	0.78^a^	0.59^a^	1				
Spirituality	0.63^a^	0.27^a^	0.33^a^	1			
Interpersonal Skills	0.85^a^	0.68^a^	0.67^a^	0.35^a^	1		
Personal Growth and Autonomy	0.85^a^	0.65^a^	0.60^a^	0.36^a^	0.76^a^	1	
Global Affect	0.57^a^	0.42^a^	0.41^a^	0.19^a^	0.42^a^	0.50^a^	1
Concurrent validity measures							
Quality of Life (EQ-5D Index)	0.14^a^	0.11^a^	0.17^a^	− 0.02	0.10^a^	0.12^a^	0.25^a^
Self-rated health (EQ-5D VAS)	0.27^a^	0.20^a^	0.22^a^	0.14^a^	0.19^a^	0.25^a^	0.30^a^
Psychological distress (K6 total score)	−0.24^a^	− 0.21^b^	− 0.22^a^	0.01	−0.18^a^	− 0.25^a^	− 0.42^a^

^a^Correlations are significant at the 0.01 level (2-tailed)

^b^Correlations are significant at the 0.001 level (2-tailed)

EQ-5D Index: Generic health state score derived from five dimensions of health related quality of life (mobility, self-care, usual activities, pain/discomfort and anxiety/depression)

EQ-5D VAS: Self-rated health score obtained from a visual analogue scale with endpoints ranging from 100 for ‘the best health you can imagine’ to 0 for ‘the worst health you can imagine’

Table 3 shows the floor and ceiling effects observed for the total PMH and subscales. Floor effect was extremely low for all domains except for spirituality while ceiling effect ranged from 9.5% to 15.3% across the PMH domains. Normative and percentile values for the PMH-I are presented in Tables 4 and 5, respectively. The mean (SD) total PMH score for the whole sample was 4.61 (0.8), while the score was 4.68 (0.9) for general coping, 4.87 (1.0) for emotional support, 3.73 (1.7) for spirituality, 4.86 (0.8) for interpersonal skills, 4.78 (0.9) for personal growth and autonomy, and 4.60 (0.9) for global affect (Table 4).

**Table 3:** Floor and ceiling effect for the PMH scales

	% Floor effect (% in the lowest score of 1)		% Ceiling effect (% in the highest score of 6)	
	Unweighted	Weighted	Unweighted	Weighted
Total Positive Mental Health	0.16	0.17	0.94	0.85
General coping	0.94	0.79	11.13	10.92
Emotional support	0.68	0.70	14.98	15.30
Spirituality	7.33	7.82	13.94	12.02
Interpersonal skills	0.68	0.68	10.14	10.14
Personal growth and autonomy	0.68	0.70	9.98	9.50
Global affect	0.68	0.53	11.82	10.57

**Table 4:** Population norms for the PMH-I and PMH levels by age, gender and ethnicity^@^

	Total Positive Mental Health	General coping	Emotional support	Spirituality	Interpersonal skills	Personal growth and autonomy	Global affect
	Mean (SD)	Mean (SD)	Mean (SD)	Mean (SD)	Mean (SD)	Mean (SD)	Mean (SD)
Whole population	4.61 (0.8)	4.68 (0.9)	4.87 (1.0)	3.73 (1.7)	4.86 (0.8)	4.78 (0.9)	4.60 (0.9)
Age							
18–29 y	4.48 (0.6)	4.57 (0.8)	4.96 (0.8)	3.32 (1.5)	4.75 (0.6)	4.63 (0.7)	4.49 (0.8)
30–39 y	4.58 (0.7)	4.67 (0.9)	4.90 (0.9)	3.63 (1.7) *	4.86 (0.7)	4.74 (0.8)	4.54 (0.8)
40–49 y	4.68 (0.8) ***	4.67 (1.1)	4.87 (1.0)	3.94 (1.7) ***	4.93 (0.9) **	4.85 (0.8) ***	4.65 (0.9) *
50–59 y	4.76 (0.8) ***	4.84 (1.1) **	4.81 (1.1)	4.10 (1.7) ***	4.95 (0.9) **	4.98 (0.9) ***	4.71 (0.8) **
60 y and above	4.68 (1.1) *	4.77 (1.4)	4.63 (1.4) *	4.09 (1.9) ***	4.85 (1.3)	4.86 (1.3) *	4.75 (1.0) **
Gender							
Men	4.58 (0.7)	4.65 (1.0)	4.76 (1.0)	3.60 (1.6)	4.83 (0.8)	4.81 (0.8)	4.62 (0.8)
Women	4.66 (0.8)	4.71 (1.0)	4.98 (1.0)^##^	3.88 (1.8) ^#^	4.89 (0.8)	4.76 (0.8)	4.58 (0.9)
Ethnicity							
Chinese	4.51 (0.7)	4.66 (0.9)	4.82 (0.9)	3.28 (1.5)	4.80 (0.7)	4.71 (0.8)	4.56 (0.8)
Malay	4.94 (0.8)^	4.80 (1.1)	5.03 (1.0) ^	5.05 (1.2) ^	5.04 (0.9) ^	4.90 (1.0) ^	4.78 (0.9) ^
Indian	4.79 (1.1) ^	4.57 (1.5)	4.93 (1.4)	4.70 (1.7) ^	4.92 (1.2)	4.99 (1.2) ^	4.53 (1.4)
Others	4.91 (0.8) ^	4.89 (1.0)	4.96 (1.1)	4.47 (1.6) ^	5.05 (0.9) ^	5.06 (0.9) ^	4.95 (0.8) ^

^@^All estimates presented in the table are weight adjusted to the population

*, **, ***: General linear regression analysis, *p* < 0.05, 0.01 and 0.001, ‘Age 18–29’ as reference group

^#^, ^##^: General linear regression analysis, *p* < 0.01 and 0.001, ‘Women’ as reference group

^: General linear regression analysis, *p* < 0.01, ‘Chinese’ as reference group

**Table 5:** PMH values in percentile by age, gender and ethnicity^a^

	Percentiles	Overall	Age					Gender		Ethnicity			
			18–29 y	30–39 y	40–49 y	50–59 y	60 y & abv	Female	Male	Chinese	Malay	Indian	Others
Total Positive Mental Health	10th	3.70	3.63	3.64	3.80	3.79	3.57	3.79	3.65	3.64	4.21	3.65	4.10
	25th	4.23	4.08	4.18	4.32	4.34	4.35	4.22	4.23	4.15	4.60	4.39	4.51
	50th	4.65	4.44	4.61	4.76	4.82	4.87	4.68	4.63	4.52	5.02	4.94	4.98
	75th	5.11	4.93	5.06	5.12	5.31	5.37	5.18	5.05	4.96	5.45	5.39	5.51
	90th	5.57	5.41	5.45	5.52	5.78	5.87	5.66	5.45	5.39	5.79	5.77	5.62
General coping	10th	3.44	3.44	3.56	3.33	3.44	2.56	3.33	3.44	3.44	3.44	3.00	3.89
	25th	4.11	4.00	4.11	4.11	4.33	4.22	4.11	4.11	4.11	4.44	4.00	4.56
	50th	4.89	4.67	4.78	4.89	5.00	5.00	4.89	4.89	4.78	5.00	4.78	5.00
	75th	5.33	5.11	5.33	5.33	5.78	5.89	5.44	5.33	5.33	5.56	5.44	5.44
	90th	6.00	5.67	5.89	5.89	6.00	6.00	6.00	5.89	6.00	6.00	6.00	5.89
Emotional support	10th	3.57	3.86	3.71	3.57	3.43	2.43	3.71	3.57	3.57	3.86	3.29	3.57
	25th	4.43	4.43	4.57	4.43	4.29	4.14	4.57	4.29	4.29	4.57	4.57	4.71
	50th	5.00	5.14	5.00	5.00	5.00	5.00	5.14	5.00	5.00	5.14	5.14	5.14
	75th	5.57	5.57	5.57	5.57	5.71	5.57	5.71	5.43	5.57	5.86	5.86	5.57
	90th	6.00	6.00	6.00	6.00	6.00	6.00	6.00	6.00	6.00	6.00	6.00	6.00
Spirituality	10th	1.14	1.14	1.14	1.43	1.29	1.00	1.43	1.14	1.00	3.71	2.57	1.57
	25th	2.14	1.86	2.14	2.57	2.71	2.43	2.29	2.14	1.86	4.71	4.00	3.86
	50th	4.00	3.00	3.86	4.43	4.57	4.57	4.14	3.86	3.14	5.29	5.00	5.00
	75th	5.14	4.86	5.00	5.14	5.71	5.57	5.43	5.00	4.71	6.00	5.86	5.71
	90th	6.00	5.86	6.00	6.00	6.00	6.00	6.00	5.86	5.71	6.00	6.00	6.00
Interpersonal skills	10th	4.00	4.00	4.00	4.00	3.89	3.22	4.00	4.00	3.89	4.22	3.89	4.33
	25th	4.44	4.33	4.44	4.56	4.56	4.56	4.44	4.44	4.33	4.78	4.56	4.67
	50th	4.89	4.78	4.89	5.00	5.00	5.00	5.00	4.89	4.89	5.00	5.00	5.11
	75th	5.44	5.22	5.33	5.44	5.67	5.67	5.44	5.33	5.33	5.56	5.67	5.67
	90th	6.00	5.67	5.78	5.89	6.00	6.00	6.00	5.89	5.89	6.00	6.00	5.89
Personal growth and autonomy	10th	3.80	3.70	3.80	4.00	3.90	3.60	3.80	3.90	3.70	3.90	4.00	4.10
	25th	4.30	4.10	4.30	4.40	4.60	4.60	4.20	4.40	4.20	4.40	4.50	4.70
	50th	4.90	4.70	4.80	5.00	5.00	5.00	4.80	4.90	4.80	5.00	5.00	5.20
	75th	5.40	5.10	5.30	5.30	5.70	5.80	5.30	5.40	5.20	5.60	5.80	5.60
	90th	5.90	5.60	5.90	5.80	6.00	6.00	6.00	5.90	5.80	6.00	6.00	6.00
Global affect	10th	3.50	3.50	3.50	3.50	3.75	3.75	3.50	3.50	3.50	3.50	3.25	4.00
	25th	4.00	4.00	4.00	4.25	4.25	4.50	4.00	4.25	4.00	4.25	4.00	4.75
	50th	4.75	4.50	4.75	4.75	4.75	4.75	4.75	4.75	4.75	4.75	4.75	4.75
	75th	5.00	5.00	5.00	5.25	5.00	5.25	5.00	5.00	5.00	5.50	5.25	5.50
	90th	6.00	5.75	5.75	6.00	6.00	6.00	6.00	6.00	5.75	6.00	6.00	6.00

^a^All estimates presented in the table are weight adjusted to the population

The mean PMH values increased significantly with increasing age for total PMH, with those aged 18–29 years having the lowest PMH score (4.48). Emotional support, however, decreased with age with those aged 60 years and above having significantly lower scores (4.63, *p* < 0.05) than those aged 18–29 years (4.96). While total PMH did not vary by gender, women had higher emotional support and spirituality scores than men (Table 4). Ethnic differences were also observed in the population, with Chinese participants showing lower total PMH scores than the other ethnic groups. Subscale scores were also lower among the Chinese for all the domains except for general coping. Most of the differences observed at bivariate level were also observed in the multivariable model after controlling for age, gender, ethnicity, marital status and education level (Table 6). In contrast to age, ethnicity and marital status, education levels were not significantly associated with total PMH values, but only with selected domains. Being married was associated with better interpersonal skills (compared to being never married) and global affect (compared to being separated/divorced/widowed) and higher education was associated with better social support.

**Table 6:** Socio-demographic factors associated with PMH after multivariable adjustment^a^

	Total Positive Mental Health	General coping	Emotional support	Spirituality	Interpersonal skills	Personal growth and autonomy	Global affect
	β (SE)	β (SE)	β (SE)	β (SE)	β (SE)	β (SE)	β (SE)
Age (10-year increment)	0.05 (0.09)**	0.05 (0.03)	−0.07 (0.03)*	0.22 (0.04)**	0.00 (0.02)	0.05 (0.03)*	0.06 (0.02)
Gender							
Men	−0.09 (0.02)*	−0.07 (0.06)	−0.22 (0.05)**	−0.30 (0.09)**	−0.06 (0.05)	0.05 (0.05)	0.04 (0.05)
Women	Ref	Ref	Ref	Ref	Ref	Ref	Ref
Ethnicity							
Malay	0.46 (0.06)**	0.16 (0.08)*	0.26 (0.07)**	1.82 (0.09)**	0.22 (0.06)**	0.22 (0.07)**	0.27 (0.06)**
Indian	0.28 (0.06)**	−0.08 (0.08)	0.11 (0.08)	1.41 (0.10)**	0.10 (0.06)	0.26 (0.07)**	− 0.02 (0.07)
Others	0.38 (0.09)**	0.23 (0.12)	0.10 (0.12)	1.14 (0.23)**	0.23 (0.11)*	0.31 (0.11)**	0.37 (0.09)**
Chinese	Ref	Ref	Ref	Ref	Ref	Ref	Ref
Marital status							
Never married	−0.07 (0.05)	− 0.03 (0.07)	− 0.09 (0.06)	− 0.02 (0.11)	− 0.14 (0.05)*	−0.10 (0.06)	− 0.05 (0.06)
Separated /divorced /widowed	−0.14 (0.08)*	− 0.10 (0.10)	−0.20 (0.10)	− 0.26 (0.17)	−0.12 (0.09)	− 0.02 (0.10)	−0.20 (0.09)*
Married	Ref	Ref	Ref	Ref	Ref	Ref	Ref
Education level							
Primary and below	−0.07 (0.18)	−0.06 (0.22)	− 0.19 (0.22)	0.07 (0.27)	0.05 (0.20)	−0.12 (0.21)	− 0.26 (0.16)
Secondary to Junior College	−0.03 (0.05)	0.01 (0.06)	−0.18 (0.06)*	− 0.05 (0.11)	0.06 (0.05)	− 0.04 (0.06)	−0.01 (0.06)
Vocational/ Polytechnic diploma	−0.03 (0.05)	0.01 (0.07)	−0.14 (0.07)*	−0.03 (0.12)	0.03 (0.06)	−0.03 (0.06)	− 0.02 (0.06)
University and above	Ref	Ref	Ref	Ref	Ref	Ref	Ref

^a^Linear regression analysis with PMH levels as dependent variables and age, gender, ethnicity, marital status and education level as independent variables

*p < 0.05, **p < 0.001

## Discussion

The findings of this multi-ethnic population-based study support the strong psychometric properties of the locally developed PMH-I for assessing positive mental health in Asian populations. A six-factor-higher-order structure was confirmed for assessing six different aspects of positive mental health: global coping, emotional support, spirituality, interpersonal skills, personal growth and autonomy, and global affect. Concurrent validity was shown through significant positive correlation of the total PMH score and its subscales with health-related quality of life and a negative correlation with psychological distress. Older age, female gender and non-Chinese ethnicities (Indian, Malay and other ethnicities) were associated with a significantly higher total PMH score. In contrast, marital status and educational level were not associated with the total PMH score, but were only associated with some of the PMH domains. In addition, age, gender and ethnicity-specific population norms were generated, allowing comparisons of individual PMH scores with population estimates. While there was minimal overall floor effect, a ceiling effect was observed for the PMH-I which was comparable to the ceiling effect found for other widely used health status measures such as the EQ-5D [31], SF-36 [32], WEMWBS [33] and the World Health Organization Disability Assessment Schedule 2.0 (WHODAS 2.0) [34].

Prior evidence on the structure of the PMH-I was based on exploratory and confirmatory factor analyses in a smaller community sample and among mental health service users in Singapore [21, 26]. In accordance with its original multi-dimensional structure, the six-factor-higher-order structure of the PMH-I in the current representative sample of multi-ethnic Singapore residents could be replicated with GOF indices close to the cut-offs recommended for a superior model fit [30]. The scale has consistently fulfilled thresholds for GOF indices in the previous studies, with CFI between 0.95–0.96, TLI of 0.95–0.96 and a RMSEA of 0.05–0.07, which were further confirmed in the current representative sample. When compared to the RMSEA of 0.076 observed in mental health service users, the present study indicated an improvement, with RMSEA value being closer to the ideal of 0.05 (Table 2). RMSEA has been reported to be sensitive to sample size, with large samples showing better RMSEA [35, 36], which could partly explain this observation. However, since RMSEA tests the model fit to the covariance matrix, it is also possible that PMH-I data fit well to the general population sample given that it comprised a wider range of PMH scores compared to the clinical population of service users [37]. A review by Chen et al. [36] concluded that RMSEA ‘depends on the structure and size of the model in complex ways that are further confounded by sample size effects’, thus highlighting the importance of using other GOF measures. This study provided generalizable estimates of GOF for the PMH-I and met fit criteria for all the four indices. Results thus demonstrate that participants clearly differentiated between the six aspects of PMH and indicate that each domain is an independent source of PMH in the population. Results also highlight that mental health and well-being scales that fail to consider independent dimensions of PMH may miss important information needed for its measurement and development of interventions.

In terms of concurrent validity, the PMH-I fulfilled convergent and discriminant expectations, showing significant positive correlation with HRQoL and negative correlation with psychological distress (Table 2). The relation between PMH and HRQoL has been previously established in the Singapore population in a smaller convenience sample [21]. The current results in the representative sample further verify these findings and add to the extant literature on well-being. In addition, for the first time, the PMH-I was shown to be associated with K6 distress scores. This concurs with observed relationship between PMH and severity of depression and anxiety in earlier studies [21, 38, 39]. The widely-used K6 distress scale screens for severe mental illnesses and has been employed in several epidemiological case-finding and interventional studies world-wide [40, 41]. K6 has also been suggested as a useful screening tool for assessing mental health risks in primary care [29]. Findings from the current study demonstrate the importance of evaluating the association between distress and PMH to identify PMH components that can moderate the risk of mental distress at a population level. Results also provide traction for drawing international comparisons of these associations to enable the development of appropriate mental health interventions. Slade [42] proposed that ‘collation of evidence from narratives of recovery from mental illness’ and their relation with mental well-being can ‘provide a counter-balance to the traditional focus of mental health services’ that relies on deficits, and these could provide a basis for mental health promotion beyond just treatment of mental illness. The hypothesis that some domains of positive mental health may influence vulnerability to psychopathology needs to be properly investigated.

The present work provides normative values based on a representative population sample in Singapore. PMH norm levels varied by age, gender and ethnicity; weighted estimates for these were established in the study which allow for comparisons between the important population groups. Previous research in smaller samples has mostly showed consistent association of PMH with age, gender and ethnicity [43, 44]. However, the normative values in this representative general population (Table 4) were slightly higher than those reported in an earlier smaller population sample and the clinical sample of mental health service users. For example the mean total PMH observed in the earlier smaller convenience sample was 4.53 ± SD 0.74 [43] while that in service users was 3.93 ± 0.95 [44] versus the weighted mean of 4.61 ± 0.80 in this representative population. It is possible that the participants in the SH-2 cohort had better mental health than the earlier samples. Individual differences in PMH have been attributed to factors such as education, race, gender, genetics and lifestyle behaviours [3] which could have influenced the norms. The focus of this national study was to provide normative data for the basic socio-demographic groups, namely, age, gender and ethnicity. Future studies designed to establish PMH norms in other sub-groups such as the employed or individuals with chronic medical conditions would be beneficial in gauging their mental health status and informing policy for mental health promotion in these groups. Growing evidence indicates that employees’ mental health influences productivity losses, including increased absenteeism, attrition and suboptimal performance at work [45, 46]. Likewise, mental well-being in individuals with chronic illnesses such as diabetes has been associated with resilience and improved clinical and social outcomes [47, 48]. The normative scores presented in this paper can be used to study improvements or decline in PMH among national samples in Singapore, and understand how PMH is associated with a variety of health, economic, and social outcomes. PMH profiles may also be studied by monitoring temporal changes in the norms across different PMH domains which could serve valuable in developing appropriate mental health promotion strategies and interventions. Results of this study thus present several advantages to researchers and policy makers.

The strength of this study lies in its sample, drawn from a survey representative of the Singapore population in terms of age, gender and ethnicity. As a novel aspect, the study also included participants belonging to other ethnic groups and those aged below 21 and over 65 which were not investigated in the past. The current study sample was derived using a national sampling frame and weight adjusted for non-response, lending support to the reliability of these estimates. Besides these strengths, some limitations need to be acknowledged. First of all, an important limitation of the study is its low response rate of 35% potentially limiting the generalizability of the study findings. The age, gender and ethnic breakdown of the sample was, however, very close to the characteristics of the Singapore population (Table 1) and likely to represent estimates applicable to these sub groups in the local population. Secondly, the cross-sectional design of the study does not allow for drawing causal inferences. Thirdly, given that the present study was designed as a secondary investigation nested within an existing cohort, the retest for reliability analyses could not be included a priori. Due to the availability of the PMH-I in English language alone, residents who were not literate in English were excluded. Irrespective of the high English literacy in Singapore (80% in population aged over 15 years) [49], this exclusion could have influenced some of the results. In addition, a likely critique of this work could relate to the multi-ethnic nature and Singapore-specific ethnic composition. Varying ethnic compositions in other populations could influence some of the results, particularly the normative values in the overall general population which should be considered while interpreting the study findings. However, we observed that the factorial structure of the PHM-I and its subscales was essentially the same when the three major ethnic groups in our study were analysed separately. Therefore, we expect that the psychometric properties of the tool will apply to homogeneous or heterogeneous populations with Chinese, Malays and/or Indians regardless of their composition. However, given that our study population resides in a high income, urban setting, it will be of value to evaluate the properties of PMH-I in other types of settings. Research should likewise focus on other samples such as those with serious health conditions, children and the oldest old to cover the full spectrum of the population. Lastly, longitudinal studies are needed to evaluate the stability of the PMH-I and thus the construct, and further investigate the influence of time changing characteristics and life events on the PMH-I performance.

## Conclusions

The assessment of positive mental health and its associated factors in clinical and non-clinical samples is important for understanding the mechanisms by which important factors and life events may influence the level of positive mental health and progression of adverse psychological outcomes in individuals and populations. The results of this study provide evidence of the strong psychometric properties of the PMH-I in a multi-ethnic Asian sample and support its use in the measurement of positive mental health in population-wide epidemiological studies. The current study provides traction for further longitudinal research on determinants of positive mental health, and for assessing the efficacy and effectiveness of interventions for mental health promotion in the population and in Asian populations outside of Singapore.

## Additional file

Additional file 1 Positive Mental Health Instrument and factor structure in the Chinese, Malay and Indian populations in Singapore. (ZIP 205 kb)

## Abbreviations

CAPI: Computer-assisted personal interviewing
CFA: Confirmatory factor analysis
CFI: Comparative fit index
EQ-5D 5 L: European Quality of Life-5 Dimensions 5 levels
GOF: Goodness-of-fit
HRQoL: Health-related quality of life
K6: Kessler 6 Psychological Distress scale
NUS-IRB: National University of Singapore Institutional Review Board
PMH: Positive mental health
PMH-I: Positive Mental Health Instrument
RMSEA: Root mean square error of approximation
SF-36: Short Form-36
SH-2: Singapore Health-2 study
SRMR: Standardized root mean square residual
TLI: Tucker-Lewis index
VAS: Visual analogue scale
WEE: Weights for enumeration exercise
WEMWBS: Warwick-Edinburgh Mental Well-Being Scale
WHO: World Health Organization
WHODAS: World Health Organization Disability Assessment Schedule
WPS: Post-stratification weights
WSF: Weights for enumeration exercise study fieldwork
