# Supplementary material for: Psychometric properties and population norms of the positive mental health instrument in a representative multi-ethnic Asian population
Source: BMC Med Res Methodol. 2018 Mar 15;18:29. doi: 10.1186/s12874-018-0487-9 (PMC5856373; doi:10.1186/s12874-018-0487-9)
Supplement: Supplementary file 1 — Positive Mental Health Instrument and factor structure in the Chinese, Malay and Indian populations in Singapore. (ZIP 205 kb) [file 12874_2018_487_MOESM1_ESM.zip › Additional file Figure 1 a-c.docx]

**Additional file Figure 1a:** **Factor structure of PMH-I in the Chinese population in Singapore (n=1149)**


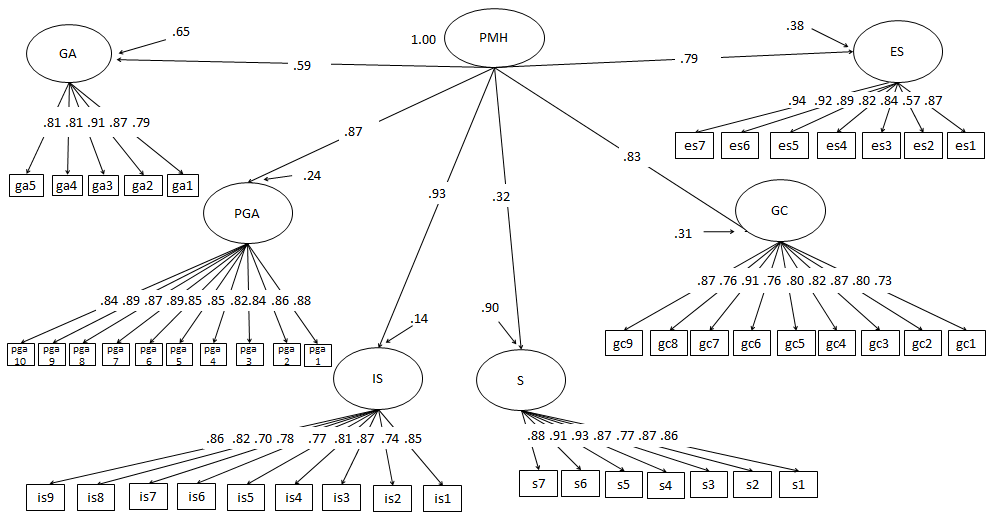


**PMH : Positive Mental Health ES : Emotional Support IS : Interpersonal Skills GA: Global Affect**

**GC : General Coping S : Spirituality PGA : Personal Growth and Autonomy**

**^#^** Item information is available in Supplementary table 1

**Additional file Figure 1b:** **Factor structure of PMH-I in the Malay population in Singapore (n=320)**


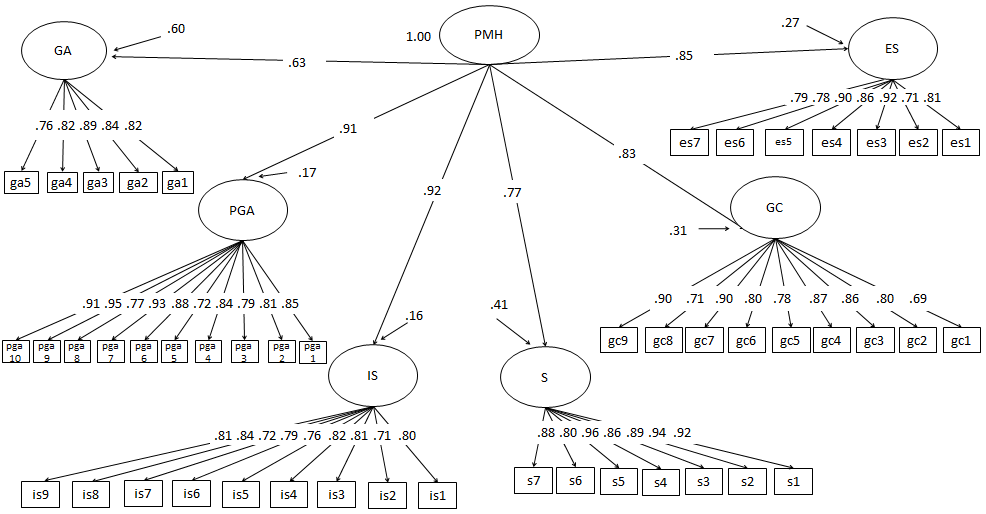
 **PMH : Positive Mental Health ES : Emotional Support IS : Interpersonal Skills GA: Global Affect**

**GC : General Coping S : Spirituality PGA : Personal Growth and Autonomy**

**^#^** Item information is available in Supplementary table 1

**Additional file Figure 1c:** **Factor structure of PMH-I in the Indian population in Singapore (n=366)**


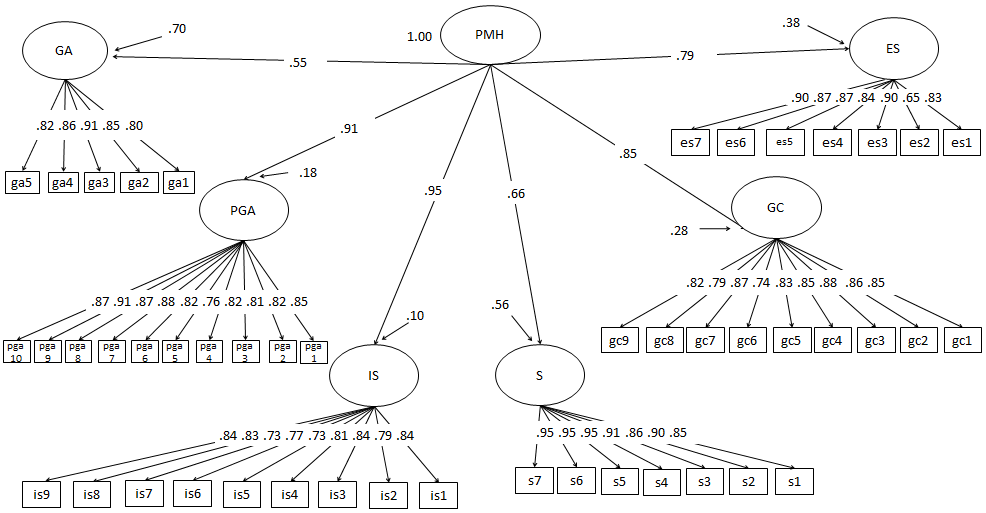
 **PMH : Positive Mental Health ES : Emotional Support IS : Interpersonal Skills GA: Global Affect**

**GC : General Coping S : Spirituality PGA : Personal Growth and Autonomy**

**^#^** Item information is available in Supplementary table 1
