# Supplementary material for: Psychometric properties and population norms of the positive mental health instrument in a representative multi-ethnic Asian population
Source: BMC Med Res Methodol. 2018 Mar 15;18:29. doi: 10.1186/s12874-018-0487-9 (PMC5856373; doi:10.1186/s12874-018-0487-9)
Supplement: Supplementary file 1 — Positive Mental Health Instrument and factor structure in the Chinese, Malay and Indian populations in Singapore. (ZIP 205 kb) [file 12874_2018_487_MOESM1_ESM.zip › Additional file Table 1.docx]

Additional file Table 1: PMH-I items^©^

|  | **General coping** |
| --- | --- |
| GC1 | I try to move on |
| GC2 | I try not to let it bother me |
| GC3 | I tell myself that things would get better |
| GC4 | I try to relax |
| GC5 | I try not to take it too seriously |
| GC6 | I do something to get my mind off the situation |
| GC7 | I try to see it in a positive light |
| GC8 | I try to see the humorous side of the situation |
| GC9 | I try to solve the problem one step at a time |
|  | **Emotional support** |
| ES1 | I spend time with people I like |
| ES2 | I try to get emotional support from family and friends |
| ES3 | I have people in my life who give me support |
| ES4 | I have a close family |
| ES5 | When I have a problem there is someone I can go to for advice |
| ES6 | There is someone to cheer me up if I am having a bad day |
| ES7 | When I am in a difficult situation there is someone I can rely on |
|  | **Spirituality** |
| S1 | I find comfort in my religion or spiritual beliefs |
| S2 | I believe God has a plan for me |
| S3 | I set aside time for meditation or prayer |
| S4 | I believe there is a higher being who looks after me |
| S5 | I feel God's presence in my life |
| S6 | I gain spiritual strength by trusting in a higher power |
| S7 | My religious beliefs influence the way I live |
|  | **Interpersonal skills** |
| IS1 | I get along well with others |
| IS2 | I make friends early |
| IS3 | I make an effort to help others |
| IS4 | I try to accept people as they are |
| IS5 | I am willing to compromise with people |
| IS6 | I try to be patient with others |
| IS7 | I am willing to give up something if it makes my family or friends happy |
| IS8 | I have no trouble keeping friends |
| IS9 | I am willing to share my time with others |
|  | **Personal growth and autonomy** |
| PGA1 | I have confidence in the decisions I make |
| PGA2 | I feel comfortable expressing my opinions |
| PGA3 | I am able to control many situations around me |
| PGA4 | I have freedom to make choices that concern my future |
| PGA5 | I feel in control of my life |
| PGA6 | I work hard to achieve my goals |
| PGA7 | I am clear about what I want in life |
| PGA8 | I am able to solve my own problems |
| PGA9 | I am focused on what I want to do in life |
| PGA10 | I know what I need to do to reach my goals |
|  | **Global affect** |
| GA1 | Calm |
| GA2 | Happy |
| GA3 | Peaceful |
| GA4 | Relaxed |
| GA5 | Enthusiastic |

© Institute of Mental Health
